# Progress in Medicine

**Published:** 1897-07-03

**Authors:** 


### Progress in Medicine.

### DISEASES OF THE DIGESTIVE ORGANS.

A melancholy interest attaches to a valuable paper on the prediagnostic treatment in grave abdominal disease. The writer, Mr. J. Greig Smith,[1] one of the first pioneers of abdominal surgery, and probably the leading writer in this country on the subject, passed away from pneumonia within two months of the date of the paper.

Not always, he remarked, can acute abdominal disease be diagnosed off hand, but, if thirsty, the patient is often given ice to suck, and, if suffering, morphia to relieve the pain. Yet, if surgical intervention should be required, some sort of diagnosis is needed, and this is impossible if the patient is narcotised; nor are the chances of success increased if liquids have been poured into a perforated stomach, or the intestines have been started into furious action by iced drinks, or paralysed by morphia injected.

"Suppose a patient, then, in ordinary health up to the moment of attack. The physician finds him in bed suffering great pain, and with the ordinary symptoms of serious abdominal disease. It may be impossible to say whether it is simply colic, intestinal, renal or biliary, or some grave condition, such as obstruction or perforation of a viscus, or even extravasation of blood."

He advises that an ounce of brandy be administered per rectum in three ounces of milk, a digital examination being made at the same time. A hot blanket should be wrapped around each leg, one drawn under the back, and hot-water bottles should be placed round him. A general and marked improvement will then be seen, and meanwhile the patient should be watched minutely, and the physical signs noted carefully. In colic the patient rolls about. He is in acute pain, and he lets everyone know it. In perforation he keeps the body still, only moving his arms and rolling his head from side to side. Intestinal strangulation presents symptoms lying between the two, but has special signs of its own. If the intestinal muscles are acting violently there is no perforation, for this is quickly followed by paralysis. The parietes may show distension, hardness from reflex or voluntary spasm, or from physical stretching. When the liver dulness has disappeared, provided there is not much distension, there is almost certainly perforation. Auscultation, too, will reveal natural diffuse sounds in colic, exaggerated rushing local ones in obstruction, but a general silence with rare blowing sounds in perforation. A few whiffs of chloroform will give relief in colic, and the patient inhales it gladly, while in perforation or strangulation he is not greedy to inhale it—it may increase the nausea, and it produces little improvement in the symptoms. Thus, as the shock passes off, the natural evolution of signs to the sight, touch, and hearing slowly takes place, and a diagnosis may be made preparatory to surgical or other treatment.

**Stomach.**—Widal and Meslay[2] report the discovery of a gastric ulcer in a patient who died from staphylococcic pyæmia. The ulcer presented perpendicular edges, and had for its base the subperitoneal adipose tissue. They consider that it was first formed by microbial action, and then enlarged by the gastric juice. Such ulcers have been found in similar septic cases and in animals killed by experimental infection. Bacteria may be absent in the ulcers from the removal by auto-digestion of the necrotic area. No symptoms of ulcer were observed during life in this case. The diagnosis of cancer may sometimes be made from the washings of the stomach, though no form of cell is peculiar to cancer. Reinebott[3] would only regard all nests as worthy of notice if repeatedly found and if a tumour can be felt; but, on the other hand, particles of tumour growth are important even when no tumour can be felt. He has searched the stomach washings in twenty-eight cases for such fragments embedded in blood clots, and found occasionally definite evidence of cancer.

Goupault[4] considers that hyper-acidity is an expression of the hyper-activity of all the stomach functions, motor activity and pyloric spasm being usually added, from which arise functional disturbances such as eructations and even dilatation of the stomach. Hence the treatment should be complete rest, at first nutrient enemata, and then very easily-digested food with sedatives. J. Thomson[5] describes a form of congenital gastric spasm. The children are born in perfect health at full time, but within a few days or weeks incessant vomiting sets in, terminated by death. The pylorus proves to be enormously hypertrophied, while except for the hypertrophy the tissues are quite normal both there and elsewhere in the body. He regards it as due to nervous disorder of the stomach and pylorus which leads to ill-co-ordinated action. The cause is, he adds, to be found in a failure of development. To estimate the motor action of the stomach, Goldsmidt[6] remarks that it is impossible to empty the stomach completely by the tube at a given time after a meal. He therefore expresses a portion of the contents, introduces 50 c.c. of distilled water, and expresses a second portion. Both specimens are filtered, and as much of the first filtrate is added to 50 c.c. of water as is necessary to raise it to the specific gravity of the second filtrate. This amount will be equal to the amount of fluid left in the stomach after the first expression.

Schwardt, of Gotha,[7] argues that displacement of the stomach is only one symptom of neurasthenia, and that treatment should be directed rather to the underlying disease than to the enteroptosis, moveable kidney, or other local lesions, though these lesions in themselves tend to produce stasis of food and auto-intoxication. Thus, he gives the case of a young woman in whom these disorders were present with membranous colitis, and in whom general hydro-therapeutic measures brought about a cure of the local troubles. Moreover, he considers that auto-intoxication from enteroptosis goes on to produce compensatory or antagonistic hyper-secretion of the thyroid, and, in a word, is the cause of Grave's disease. Similar instances of compensation are, he thinks, to be found in the diarrhœa, sweating, and vomiting which occur in these neurasthenic sufferers. If this be true the thyroid endeavours to neutralise the effete matters in the blood, and in so doing produces other derangements. It is, of course, right to remark that many cases of enteroptosis are mechanically produced, and do not necessarily cause auto-intoxication, nor do they always occur in neurasthenics.

An instance of marked dilatation of the stomach caused by cicatricial contraction is recorded by Hotaling,[8] which went on to a fatal termination with no distinctive symptoms till shortly before death, when continuous pain and coffee-ground vomiting seemed to point to cancer. This diagnosis was confirmed by the presence of what seemed to be a mass of cancerous growth in the pyloric region and the yellow hue of a typical cancer patient. After death it was found that there was no cancer, and the nodular tumour turned out to be the left lobe of the liver, which was marked by ridges due to the pressure of the ribs.

The use of Taka diastase is recommended by C. C. Fite,[9] amongst others, who brings out the fact that it not only breaks up the starches into maltose, but also disintegrates some of the albumens in the stomach contents forming albumoses and peptones. Even apart from this power, the breaking-up and conversion of carbohydrates renders easier gastric digestion of albuminoids. He also proposes to add taka to the barley-water with which milk for infants is diluted. The conversion of starches, indeed, in itself depends much upon the variety of the starch employed. Stone[10] finds that some of those derived from common articles of food require as much as eighty times the period in which others are converted. The potato is one of the most quickly changed; while, in the process of baking, the starch of cereals is partially broken down and made more quickly convertible.

**Intestines.**—Chronic fæcal retention may be suspected from various symptoms, apart from local signs. Thus, Herschell[11] mentions mental distress, irritability of temper, a bitter taste in the mouth, especially when coupled with progressive emaciation, sallowness and pigment spots on the skin of the arms, itching rashes, such as urticaria, and a red ring formed in the urine on the addition of nitric acid. The latter, though not an invariable test, is of some importance, occurring in 15 out of 32 cases examined by the author. It is found, indeed, in the pale urine of anæmia, whether the dyspepsia and fæcal accumulation be a result, or, as Sir Andrew Clark believed, a cause of the anæmia. For the removal of these accumulations, he recommends in difficult cases a powerful solvent consisting of an injection of two ounces of fresh ox-gall in half a pint of warm olive oil every night, and retained as long as possible. This liquefies the hardened masses very rapidly. After the rectum is completely cleared, high injections of hot water containing boric acid to the amount of half a gallon should be introduced. Injection should not be made by a syringe, but by a continuous pressure not exceeding 1½ to 2 lbs. to the inch. An antiseptic should always be added, otherwise the solution of old fæcal matter may cause auto-intoxication. The hips should be raised, and the water introduced very gently, while the operator's hand is placed on the abdomen to note the rapidity of distension by the fluid. With reference to the use of these "high" injections, we may notice that Herz[12] finds there is incompetency of the ileocæcal valve in many cardiac and alcoholic subjects, and those suffering from chronic affections of the large intestines. If the ascending colon in these patients be compressed with the edge of the left hand, and the cæcum be grasped with the right, the contents of the cæcum, he thinks, will be heard to pass back into the ileum with a gurgling sound. Much constipation and loss of flesh accompany the condition; purgatives, disinfectants, and especially massage are of some value, but ectropion of the valve is incurable. Idiopathic dilatation of the colon and sigmoid flexure has been occasionally observed, and nearly all the cases have proved fatal. There is always constipation, notable distension, pain, vomiting, and intermittent liquid stools. Martin[13] remarks that treatment is most unsatisfactory; neither purgatives, massage, nor operative measures have been of any use. The disease may occur at birth or develop during later life. It is most frequent in males. The cause seems to be purely mechanical, and has been ascribed to various conditions, such as abnormal development of the parts or adhesions. Of course, we must exclude from the cases those where rectal constriction exists, and the dilatation of the colon should be verified by a careful physical examination.

[1] Treatment, March 11. [2] Med. Week, March 19. [3] Med. Chron., Feb. [4] Med. Week, Feb. 19. [5] Brit. Med. Jour., May 15. [6] Brit. Med. Jour., May 18. [7] Lancet, May 1. [8] Med. Record, March 20. [9] Med. Bulletin. [10] United States Depart. of Agriculture Bulletin, 34. [11] Med. Press and Circ., April 21. [12] Med. Week, April 22. [13] Med. Chron., March.

